# Assessing Strength Asymmetries with Rotational Inertial Technology: Exercise-Specific Patterns and Temporal Changes in Professional Male Soccer Players

**DOI:** 10.3390/sports14040145

**Published:** 2026-04-09

**Authors:** Alvaro Murillo-Ortiz, Javier Raya-Gonzalez, Moises Falces-Prieto, Samuel Lopez-Mariscal, Francisco Javier Iglesias-Garcia, Luis Manuel Martinez-Aranda

**Affiliations:** 1 Research Group on Sport and Physical Education for Personal and Social Development (GIDEPSO), Department of Specific Didactics, Faculty of Education Sciences and Psychology, University of Cordoba, 14071 Cordoba, Spain; z22muora@uco.es; 2 GIR07, Research Group in Physical Activity and Sports Sciences, University Isabel I, 09003 Burgos, Spain; mfalpri@gmail.com; 3 High Performance Department KMSK Deinze, 9800 Deinze, Belgium; samuellopm@gmail.com (S.L.-M.); javi.iglesias30@gmail.com (F.J.I.-G.); 4 Department of Sports and Computer Sciences, Faculty of Sports Sciences, Universidad Pablo de Olavide, 41089 Seville, Spain; 5 Research Group CTS563, Faculty of Education, University of Malaga, 29010 Malaga, Spain; 6 Science-Based Training Research Group (SEJ-680), Physical Performance and Sports Research Center, Universidad Pablo de Olavide, 41089 Seville, Spain

**Keywords:** eccentric, flywheel, resistance exercise, asymmetry, football, soccer players, monitoring

## Abstract

Inter-limb asymmetries are common in soccer players and are frequently monitored in high-performance settings; however, their expression across different flywheel-based strength exercises, movement phases, and over time remains unclear. This study aimed to (i) compare inter-limb power asymmetry magnitudes across multiple unilateral flywheel exercises and between concentric (CON) and eccentric (ECC) phases, and (ii) describe changes in these asymmetries over an 8-week period of routine soccer training, stratified by baseline asymmetry magnitude. The present study was designed as an observational and descriptive study. Twenty-one professional male soccer players completed two testing sessions separated by eight weeks. Players performed six unilateral flywheel exercises targeting hip- and knee-dominant quadriceps (Qhip, Qknee), hip- and knee-dominant hamstrings (Hhip, Hknee), adductors (ADD), and abductors (ABD). For each exercise and limb, the repetition with the highest CON mean power and its consecutive ECC phase were selected for analysis. Inter-limb asymmetry (%) was calculated for mean and peak power in both phases. Across exercises, ECC asymmetries were generally greater than CON asymmetries, with the largest values observed for Qknee peak power (CON: 12.86 ± 11.04%; ECC: 27.60 ± 13.65%) and Hknee peak power (CON: 10.45 ± 11.26%; ECC: 24.01 ± 20.46%). Exercise-specific patterns were evident, with generally weak associations between asymmetries across tasks. Over time, players classified with higher baseline asymmetry (≥10%) presented lower values at follow-up in several outcomes (particularly ECC-related measures), whereas players with lower baseline asymmetry (<10%) showed small increases or remained stable; These between-group patterns should be interpreted cautiously, as they may be more strongly influenced by regression to the mean and measurement variability than by underlying physiological changes. Overall, inter-limb power asymmetries assessed with flywheel technology were phase- and exercise-specific in this professional soccer sample. These descriptive findings may help contextualize phase-specific and multi-exercise asymmetry monitoring in professional soccer settings.

## 1. Introduction

Inter-limb asymmetry is an inherent characteristic of human movement, arising from both anatomical factors and functional lateralization of the neuromuscular system [1]. In soccer, this asymmetry may be further accentuated by sport-specific demands, such as preferential use of one limb for kicking, cutting, and rapid changes of direction, which can lead to unequal mechanical loading and neuromuscular development between limbs [2,3]. While a certain degree of asymmetry may reflect functional specialization, excessive or poorly managed inter-limb differences have been proposed as a potential concern in high-performance sport contexts [4]. On the other hand, the relationship between asymmetries and an increased risk of injury remains inconsistent, although previous studies have shown that inter-limb asymmetries have been associated with poorer performance [5], making their detailed assessment particularly relevant in elite sport.

Inter-limb asymmetries have been widely investigated using functional performance tasks such as jumping, sprinting, or change-of-direction tests [6]. In strength assessments, however, much of the existing literature has relied on traditional testing methods that do not clearly differentiate between concentric (CON) and eccentric (ECC) muscle actions [7,8]. This distinction may be particularly relevant, as eccentric actions are characterized by greater force demands and distinct neural control strategies compared with concentric actions [9,10], which are key determinants of both performance and injury risk in soccer [11]. Rotational inertial devices enable the independent assessment of concentric (CON) and eccentric (ECC) phases and have demonstrated sensitivity for detecting between-limb mechanical differences during unilateral strength exercises [12]; however, findings derived from these tasks may not generalize across different movement patterns.

Modern soccer places increasingly high physical demands on players, with evidence of a progressive rise in high-intensity actions such as sprinting, changes of direction, and jumping [13,14]. This justifies the need to include neuromuscular monitoring in soccer routines. Despite growing interest in inter-limb asymmetry monitoring, several important limitations remain in the current literature. First, most previous studies have evaluated asymmetries using single tasks or isolated movement patterns, limiting understanding of how asymmetries vary across different exercises targeting the same or related muscle groups [15,16]. Second, relatively few studies have examined asymmetries separately during the CON and ECC phases using flywheel technology, despite the widespread use of eccentric overload training in elite soccer environments [11,17]. Third, longitudinal data describing how inter-limb asymmetries fluctuate over time in response to routine training exposure, particularly when players are stratified according to baseline asymmetry magnitude, are scarce.

Inter-limb asymmetries are often discussed in relation to injury risk and performance outcomes. However, the available evidence remains inconsistent, and causal relationships have not been clearly established [16,18]. As such, a detailed descriptive characterization of asymmetry magnitude, phase specificity, and exercise dependency may provide valuable context for interpreting asymmetry data collected in professional soccer settings, without assuming direct effects on injury or performance. Therefore, the aims of this study were to: (i) compare inter-limb power asymmetry magnitudes across multiple unilateral flywheel-based strength exercises and between CON and ECC phases; and (ii) describe changes in these asymmetries over an 8-week period of routine soccer training, stratified according to baseline asymmetry level. Based on previous evidence, it was expected that eccentric-phase asymmetries would generally be greater than concentric-phase asymmetries and that asymmetry patterns would be exercise-specific. No causal effects of training were hypothesized due to the observational nature of the study.

## 2. Materials and Methods

### 2.1. Participants

Twenty-one male professional soccer players (age: 26.3 ± 5.1 years; height: 182.3 ± 0.6 cm; body mass: 75.9 ± 5.9 kg) from the same team competing in the Belgian second division (Challenger Pro League) voluntarily participated in this study. All players were free from musculoskeletal injury and had at least five years (8 ± 3 years) of competitive experience. The distribution of playing positions was as follows: 5 central defenders, 4 fullbacks, 4 midfielders, 4 wingers, and 4 forwards, with 13 players classified as right-limb dominant and 8 as left-limb dominant. Inclusion criteria required completion of both assessment sessions (pre-season and follow-up) and all prescribed exercise repetitions. Players who sustained an injury during the follow-up period were excluded from the final analysis. The study was approved by the Ethics Committee of the University of Córdoba (Code: CEIH-24-50; 20 November 2024) and conducted in accordance with the Declaration of Helsinki. All participants provided written informed consent before participation.

### 2.2. Study Design

This observational longitudinal study aimed to describe lower-limb inter-limb power asymmetries across six unilateral flywheel (rotational inertia) exercises and to examine their temporal evolution over an 8-week period in professional soccer players. Two testing sessions were conducted: the first at the end of the second week of the preseason, and the second eight weeks later. Between testing sessions, players followed their regular team training schedule, which included five on-field training sessions per week, two to three gym-based strength sessions, and one to two official or friendly matches per week. This training exposure was considered representative of typical physical demands in professional soccer [15,19]. Inter-limb asymmetries were analyzed for mean and peak power outputs during both the concentric (CON) and eccentric (ECC) phases of each exercise. To explore temporal patterns, players were stratified into low- and high-asymmetry groups based on baseline asymmetry magnitude for each exercise and variable, and pre- to post-assessment comparisons were subsequently performed. Each player completed two sets of eight repetitions per limb for each exercise, with exercise order randomized. Testing sessions were conducted between 9:00 and 11:00 a.m. under stable environmental conditions (17–22 °C; 60–70% relative humidity). Prior to testing, participants performed a standardized warm-up consisting of joint mobility exercises, dynamic stretching, and submaximal repetitions of the tested exercises.

### 2.3. Procedures

Six unilateral exercises were evaluated on a rotational inertia device (Pulley Pro C3, Proinertial^®^, Barcelona, Spain): (1) Knee flexion (Hknee); (2) Knee extension (Qknee); (3) Hip flexion (Qhip); (4) Hip extension (Hhip); (5) Hip abduction (ABD); (6) Hip adduction (ADD). All exercises were performed in a unilateral manner for both limbs. A single inertial load of 0.0335 kg·m^2^ was used for all exercises and remained constant across testing sessions. This load was selected based on the team’s habitual training practices and previous experience with flywheel-based resistance training. All participants were familiar with the six exercises, as they were routinely included in their regular strength-training program. Therefore, no additional familiarization sessions were conducted specifically for the purposes of this study. For all exercises, participants were instructed to perform the CON phase (i.e., from 90° knee flexion to full extension) as fast as possible and to actively decelerate the flywheel during the final portion of the eccentric phase in order to control the return movement. This execution strategy is consistent with current recommendations for flywheel resistance exercise performance [17,20]. No real-time feedback regarding power output or inter-limb asymmetry was provided during testing.

The exercises performed by the subjects during the study are described in detail below:

Qhip: Participants started in a standing position with the hips in neutral alignment, knees slightly flexed (~10°), and feet shoulder-width apart. From this position, they performed a unilateral hip flexion to ~10° of hyperextension against the inertial load, followed by a controlled eccentric return to the starting position.

Qknee: Participants were positioned prone with the hips in neutral alignment, the knee actively flexed to 90°, and the ankle in neutral (0° dorsiflexion). From there, they performed a unilateral knee extension from 90° flexion to full extension. After reaching maximal extension, they were instructed to actively decelerate the return phase until the starting position was regained.

Hhip: Participants lay supine with the hips flexed to 60°, knees fully extended, and the ankles secured with pads connected to the flywheel device. The task consisted of unilateral hamstring action through hip extension from 60° flexion to neutral, followed by a controlled eccentric phase back to the starting position.

Hknee: Players were positioned prone with the hips in neutral alignment, knees extended, and ankles secured to the flywheel device. From this position, they performed unilateral knee flexion from 0° to 120° against inertial resistance, followed by a controlled eccentric return to the starting position.

ADD: Participants lay supine with the hips in neutral alignment (0° flexion/extension), thighs abducted to 30°, and knees extended. The ankles were secured with pads attached to the flywheel device. They then performed unilateral hip adduction from 30° abduction to neutral, followed by a controlled eccentric phase.

ABD: Players were positioned supine with the hips in neutral alignment, thighs at 10° of adduction, and knees extended, with the ankles secured to the flywheel device. From this position, they performed unilateral hip abduction from 10° adduction to 45° abduction, ending with a controlled eccentric return to the starting position.

Mechanical data were collected using a rotational encoder and processed with Chronojump software (Chronojump^®^, Barcelona, Spain). This device has been used previously in similar studies [21,22], and a recent systematic review has supported its validity and reliability for assessing physical performance and asymmetries [23]. Instantaneous power values were recorded separately for the concentric and eccentric phases of each repetition. Mean power was calculated as the average of all instantaneous power values across the full range of motion of each phase, whereas peak power was defined as the highest instantaneous power value achieved within that same phase. For each exercise and limb, the repetition with the highest concentric mean power and its immediately subsequent eccentric phase were selected for analysis, in line with previous studies [24,25]. A total of two sets of eight repetitions were performed, with 4 min of recovery between sets. The first set was used for familiarization and warm-up purposes, whereas the second set was used for data analysis. From this second set, the repetition selected for analysis was the one that showed the highest mean concentric power, considering the instantaneous values recorded during the movement and its immediately subsequent eccentric phase.

### 2.4. Asymmetry Calculation

Inter-limb asymmetry magnitude was calculated for all unilateral variables following previously described methodological approaches [4,26]. For each exercise and testing session, the repetition selected for analysis was the repetition from the second set that exhibited the highest concentric mean power value. This repetition and its immediately subsequent eccentric phase were used as the reference for all power variables.

For each exercise, phase, and testing session, the limb that produced the higher concentric mean power value in the selected repetition was defined as the stronger limb, whereas the contralateral limb was defined as the weaker limb. Asymmetry magnitude was then expressed as the relative percentage difference between limbs using the following equation:

$$Asymmetry\;(\%)=\frac{\left(Stronger\;limb-Weaker\;limb\right)\times 100}{Stronger\;limb}$$

This approach was chosen to standardize asymmetry calculation across variables using a consistent performance criterion and to quantify the magnitude of between-limb differences independently of limb dominance, as commonly applied in the asymmetry literature [4,26]. Asymmetry values were calculated separately for mean and peak power during both the concentric (CON) and eccentric (ECC) phases of each exercise.

### 2.5. Statistical Analysis

Data are presented as mean ± standard deviation (SD). Normality of distribution was assessed using the Shapiro–Wilk test. Inter-limb asymmetries were calculated for all unilateral variables following the same methodological approach described in the previous literature [27]. For each test, the limb that produced the higher value was defined as the stronger limb, whereas the limb with the lower value was defined as the weaker limb. Asymmetry magnitude was then calculated as the relative percentage difference between limbs, using the stronger limb as the reference value, as previously described [4,26]. This approach allowed the quantification of inter-limb asymmetries independently of limb dominance, focusing instead on functional differences in mechanical performance. Asymmetry values were calculated separately for mean and peak power during both the CON and ECC phases. Differences between concentric and eccentric asymmetry values were analyzed using paired-samples *t*-tests. To examine relationships between asymmetries across exercises, Pearson’s correlation coefficients (r) were calculated. The magnitude of correlations was interpreted as trivial (<0.1), small (0.1–0.3), moderate (0.3–0.5), large (0.5–0.7), very large (0.7–0.9), or nearly perfect (>0.9) [28]. To evaluate temporal changes in asymmetry, players were stratified into low- and high-asymmetry groups based on baseline values, with asymmetries <10% categorized as low and asymmetries ≥10% categorized as high [29]. ANCOVA was used to analyze between-group differences in pre-to-post changes, whereas within-group differences were assessed using paired-samples *t*-tests. Effect sizes (Cohen’s d) were calculated and interpreted as trivial (<0.2), small (0.2–0.49), moderate (0.5–0.8), or large (>0.8). Statistical significance was set at *p* < 0.05. All analyses were performed using JASP software (v0.18.1; University of Amsterdam, The Netherlands). To control for Type I error due to multiple analysis, a Bonferroni correction was applied, and consequently, statistical significance was set at *p* < 0.003.

## 3. Results

### 3.1. Asymmetry Values by Muscle Group and Movement Phase

Differences between the CON and ECC phases are presented in Table 1, Table 2 and Table 3. In the quadriceps exercises, both mean and peak power asymmetries were greater during the ECC phase. Qknee exhibited the highest peak power asymmetry, with larger values in ECC (27.61 ± 13.65%) than in CON (12.86 ± 11.04%). Similarly, in Qhip, ECC peak power asymmetry (17.50 ± 20.08%) exceeded the CON value (10.84 ± 11.81%).

In the hamstring exercises (Table 2), higher peak power asymmetry values were observed during the eccentric (ECC) phase compared with the concentric (CON) phase, particularly for the knee-dominant hamstring exercise (Hknee). Peak power asymmetry in Hknee reached 24.01 ± 20.46% during the ECC phase (effect size = 1.204) and 10.45 ± 11.26% during the CON phase. For the hip-dominant hamstring exercise (Hhip), peak power asymmetry was also higher in the ECC phase than in the CON phase, although the differences were non-significant.

For the adductor and abductor exercises (Table 3), although not statistically significant, higher inter-limb asymmetry values were observed for the adductor exercise (ADD) compared with the abductor exercise (ABD) across most variables. In ADD, mean power asymmetry during the concentric (CON) phase reached 15.54 ± 9.55%, and peak power asymmetry during the eccentric (ECC) phase reached 17.12 ± 11.74%. In contrast, the ABD exercise showed lower asymmetry magnitudes, with mean power asymmetry values of 9.30 ± 6.20% during the CON phase and 10.62 ± 5.86% during the ECC phase.

Correlation analyses across exercises (i.e., 15 correlations within each family of comparisons) generally revealed weak and non-significant associations between inter-limb asymmetry values across the different movement patterns. These analyses should be interpreted as exploratory, given the number of comparisons performed and the absence of a priori hypotheses for specific exercise pairings. A moderate positive correlation, but non-significant, was observed for concentric (CON) peak power asymmetry between the hip-dominant and knee-dominant quadriceps exercises (Qhip and Qknee; *r* = 0.464, *p* = 0.034). In contrast, eccentric (ECC) mean power asymmetry showed a moderate negative correlation, but non-significant, between the adductor (ADD) and abductor (ABD) exercises (*r* = −0.454, *p* = 0.039), with a similar negative association also observed for ECC peak power asymmetry between these exercises (*r* = −0.489, *p* = 0.025). These findings should be interpreted with caution and do not imply mechanistic relationships, but rather indicate potential patterns that may warrant further investigation.

### 3.2. Evolution of Asymmetries Based on Initial Levels

Participants were stratified into two groups (low vs. high asymmetry) based on baseline asymmetry values for each exercise. Across muscle groups, different patterns of change were observed between testing sessions depending on baseline asymmetry level. Players classified in the high-asymmetry group tended to present lower asymmetry values at post-testing, whereas players classified in the low-asymmetry group tended to show higher values or minimal changes.

For the quadriceps exercises (Table 4), the high-asymmetry group showed lower eccentric (ECC) mean power asymmetry values at post-testing for both the hip-dominant quadriceps exercise (Qhip; 18.25 ± 6.26% to 11.54 ± 11.95%) and the knee-dominant quadriceps exercise (Qknee; 19.11 ± 5.70% to 16.11 ± 7.54%). In contrast, the low-asymmetry group displayed higher CON and ECC mean power asymmetry values at post-testing in both exercises.

Regarding the hamstring exercises (Table 5), despite the lack of statistical significance, lower eccentric (ECC) peak power asymmetry values were observed at post-testing in the high-asymmetry group for both the hip-dominant hamstring exercise (Hhip; 24.85 ± 16.67% to 17.22 ± 13.78%) and the knee-dominant hamstring exercise (Hknee; 35.61 ± 17.59% to 25.75 ± 38.78%). In contrast, the low-asymmetry group showed higher asymmetry values at post-testing across several variables, particularly for ECC mean and peak power.

In the adductor and abductor exercises (Table 6), lower asymmetry values at post-testing were observed in the high-asymmetry group for most outcomes. For example, eccentric (ECC) peak power asymmetry values were lower at post-testing for the adductor exercise (22.96 ± 9.82% to 18.89 ± 13.18%) and showed minimal change for the abductor exercise (23.04 ± 11.92% to 22.95 ± 13.01%). In contrast, the low-asymmetry group displayed higher asymmetry values at post-testing across several variables, including a marked increase in adductor concentric (CON) mean power asymmetry (6.81 ± 3.47% to 15.59 ± 10.75%). However, these descriptive differences were not statistically significant.

Results of the ANCOVA are presented in Table 7. No significant differences were observed in any between-group comparisons.

## 4. Discussion

The present study described inter-limb asymmetry patterns across several flywheel (rotational inertial) strength exercises in professional soccer players, with a specific focus on power output during the concentric (CON) and eccentric (ECC) phases. In addition, changes in asymmetry magnitude were examined over an 8-week period by stratifying players according to baseline asymmetry level (low vs. high). Three main patterns were observed: (i) asymmetry values were generally higher during the ECC phase compared with the CON phase; (ii) asymmetry magnitudes varied across exercises, with larger values observed in the adductor and quadriceps exercises; and (iii) different temporal patterns were observed depending on baseline asymmetry level, with lower post-testing values in players classified as highly asymmetric at baseline and small increases or minimal changes in those with lower initial asymmetry.

The observation of greater asymmetry magnitudes during ECC actions is consistent with previous studies reporting larger between-limb differences during eccentric compared with concentric muscle actions [20,30]. This pattern may be related to the distinct mechanical and neuromuscular characteristics of eccentric contractions, as well as to differences in familiarity with eccentric-dominant tasks. However, it should be noted that no neuromuscular mechanisms were directly assessed in the present study, and therefore these explanations remain speculative. In this context, flywheel devices, which place greater mechanical demands on the eccentric phase, may accentuate existing between-limb differences compared with traditional isotonic resistance exercises [17]. This pattern was particularly evident in the knee-dominant quadriceps exercise, which exhibited the highest eccentric peak power asymmetry.

A second relevant observation was the exercise-specific nature of inter-limb asymmetries, supporting previous evidence indicating that asymmetry magnitude in professional soccer players is often task- and muscle-group specific and does not necessarily transfer across different movement patterns [16,31]. In the present study, adductor exercises showed higher asymmetry values than abductor exercises, which is consistent with previous findings reporting frequent adductor strength imbalances in soccer players [32]. These differences may be related to the functional demands placed on the adductors during sport-specific actions such as cutting, directional changes, and kicking, although such mechanisms cannot be directly confirmed by the present data. In contrast, the hip-dominant hamstring exercise displayed comparatively lower asymmetry magnitudes across outcomes. This observation may be influenced by the frequent involvement of the hamstrings during sprinting and other high-speed running actions in soccer, potentially resulting in more symmetrical loading patterns between limbs over time [15]. However, these findings should be interpreted in light of previous flywheel-based research, which has typically assessed inter-limb asymmetries using a single exercise, most commonly the half-squat. This limits direct comparison with the present study, which included multiple exercises involving different muscle groups and movement patterns. Therefore, although the exercise-specific asymmetry patterns observed here are consistent with the broader concept of task specificity, caution is needed when comparing these results with previous flywheel-based studies. At the same time, this broader exercise selection represents a novel aspect of the present study and may help provide a more comprehensive description of inter-limb asymmetry in professional soccer players.

The moderate positive correlation observed between the hip- and knee-dominant quadriceps exercises suggests that asymmetry patterns may be partially shared across tasks involving the same primary muscle group, despite differences in joint dominance [11]. However, this association should be interpreted with caution, as it does not necessarily reflect a consistent or transferable biomechanical mechanism. Conversely, the negative associations observed between the adductor and abductor exercises, particularly during the eccentric phase, indicate that asymmetry magnitudes in these movements may vary in opposite directions. These findings should be considered exploratory, and although they may be related to differences in the functional roles of these muscle groups during lateral movements, no direct biomechanical or neuromuscular mechanisms were assessed in the present study. Therefore, any interpretation regarding underlying mechanisms remains speculative and should be approached cautiously.

Changes in inter-limb asymmetry magnitude differed according to baseline asymmetry level. Players classified as highly asymmetric at baseline generally showed lower values at post-testing, whereas those with lower initial asymmetry tended to show small increases or little change. This bidirectional pattern is consistent with regression toward the mean, which is common when participants are grouped according to extreme baseline values. In the present study, this effect may have been amplified by the subgroup classification approach. Given the observational design, the lack of a control group, and the absence of quantified training load, regression toward the mean is a plausible explanation for part of the observed changes. Therefore, these subgroup comparisons should be interpreted cautiously and should not be assumed to reflect true adaptations over time.

Repeated exposure to similar training demands may also have contributed to changes in asymmetry magnitude over time. However, the present data do not allow these potential effects to be distinguished from measurement variability or regression toward the mean. Accordingly, the changes observed in both high- and low-asymmetry groups should be interpreted cautiously and not assumed to reflect neuromuscular adaptations.

From an applied perspective, these findings highlight the importance of contextualizing longitudinal asymmetry data rather than interpreting changes in isolation. Regular monitoring of inter-limb asymmetries, particularly during eccentric actions, may assist practitioners in tracking within-player variability over time, but such data should be interpreted alongside baseline values, with particular attention to measurement error and task specificity. In this regard, small or short-term changes in asymmetry magnitude should be interpreted cautiously, as they may fall within the expected range of measurement variability rather than represent true physiological adaptations. Given the exercise-specific nature of asymmetry observed in this study, a multi-exercise assessment approach may provide a more comprehensive description of inter-limb differences than reliance on a single test.

Despite the insights provided by this study, several limitations should be acknowledged. First, the sample comprised players from a single professional soccer team, which limits the generalizability of the findings to other competitive levels, teams, or playing styles. Second, the study followed an observational longitudinal design without a control group or experimental manipulation, and training load was not quantified during the observation period. Therefore, causal inferences regarding the effects of training on inter-limb asymmetry cannot be made.

In addition, players were stratified according to baseline asymmetry magnitude, and the bidirectional pattern observed over time is consistent with regression toward the mean. Although this issue was considered in the interpretation of the results, it could not be fully controlled within the present design. From a methodological perspective, asymmetry calculations were based on a single selected repetition per exercise and session, and reliability indices such as ICC, coefficient of variation, or minimal detectable change were not established for the specific protocol used. Flywheel resistance devices inherently show repetition-to-repetition variability because performance outputs depend on the force applied during the concentric phase and the athlete’s ability to decelerate the inertia during the eccentric braking phase. Consequently, mechanical outputs may fluctuate across repetitions. Although selecting a representative repetition is common in flywheel-based assessments, this approach may increase susceptibility to within-subject variability, particularly for variables such as Qknee and Hknee, which showed the largest asymmetries in the present study.

Furthermore, asymmetry magnitude was quantified without retaining directional information (e.g., dominant vs. non-dominant limb), which limits interpretation of limb-specific changes over time. The large number of variables and comparisons analysed also increases the risk of type I error despite the statistical corrections applied. Finally, the analyses were restricted to power-related variables and did not include injury incidence, performance outcomes, or additional neuromuscular or biomechanical measures, such as force–velocity profiling or electromyography, which would be necessary to establish the functional or clinical relevance of the observed asymmetry patterns. Future studies incorporating longer follow-up periods, controlled interventions, and multidimensional outcome measures are warranted.

Future research should examine the longer-term evolution of inter-limb asymmetries using controlled experimental designs, including interventions that combine strength-based training with sport-specific technical and cognitive demands. Longitudinal studies exploring the relationship between asymmetry metrics, injury occurrence, and performance outcomes are also warranted to clarify their functional relevance in professional soccer. In addition, distinguishing task-specific asymmetry expressions from underlying strength-related asymmetries may improve the interpretation of asymmetry metrics across different assessment contexts. Expanding assessment approaches to include complementary kinetic and kinematic markers, such as those obtained through motion capture systems or force platforms, could further enhance the precision and contextual interpretation of inter-limb differences.

## 5. Conclusions

This study provides a descriptive characterization of inter-limb power asymmetry patterns in professional soccer players across multiple unilateral strength exercises performed with rotational inertial technology. The results indicate that asymmetry magnitudes were generally higher during eccentric compared with concentric phases and that the magnitude of asymmetry was strongly exercise-specific. However, these findings should be interpreted with caution due to the methodological constraints of the study, including the sample size, the absence of reliability indices, and the use of a single-repetition analysis. Therefore, while the results offer preliminary insights into asymmetry patterns, their direct practical application should be considered carefully and within the context of these limitations.

Differences in temporal patterns were observed according to baseline asymmetry level. However, these changes should be interpreted cautiously because of the observational design of the study and the potential influence of regression toward the mean and measurement variability. Overall, the findings highlight the importance of considering phase specificity, task dependency, and baseline asymmetry magnitude when interpreting inter-limb asymmetry data in professional soccer settings. Multi-exercise, phase-specific assessments may provide useful contextual information for longitudinal monitoring, without implying direct effects on injury risk or performance outcomes. These findings should therefore be considered descriptive and exploratory, and primarily useful for generating future hypotheses rather than establishing definitive conclusions.

### Highlights of Key Trends

- Inter-limb power asymmetry magnitude differed between concentric and eccentric phases during unilateral flywheel-based strength exercises.
- Eccentric phases generally exhibited higher asymmetry values than concentric phases across multiple muscle groups.
- Asymmetry magnitude was highly exercise-specific, with weak associations observed between different movement patterns.
- Different temporal patterns were observed according to baseline asymmetry level, although these changes should be interpreted cautiously.
- Multi-exercise, phase-specific assessments may help contextualize inter-limb asymmetry data in professional soccer players.

## Figures and Tables

**Table 1:** Inter-limb power asymmetries during concentric and eccentric phases in quadriceps exercises.

Variable	Exercise			
	Quadriceps Hip		Quadriceps Knee	
	CON	ECC	CON	ECC
Mean power asymmetry (%)	8.77 ± 7.74	12.07 ± 8.27 *	12.50 ± 8.09	13.97 ± 8.26
Peak power asymmetry (%)	10.84 ± 11.81	17.50 ± 20.08	12.86 ± 11.04	27.60 ± 13.65 ***

Data are presented as mean ± standard deviation (SD). CON = concentric phase; ECC = eccentric phase. * *p* < 0.05, *** *p* < 0.001 (difference between CON and ECC phases).

**Table 2:** Inter-limb power asymmetries during concentric and eccentric phases in hamstring exercises.

Variable	Exercise			
	Hamstring Hip		Hamstring Knee	
	CON	ECC	CON	ECC
Mean power asymmetry (%)	8.685 ± 6.666	10.570 ± 8.243	9.127 ± 7.040	7.996 ± 6.818
Peak power asymmetry (%)	12.480 ± 8.081	19.758 ± 17.241	10.449 ± 11.255	24.010 ± 20.463 *

Data are presented as mean ± standard deviation (SD). CON = concentric phase; ECC = eccentric phase. * *p* < 0.05 (difference between CON and ECC phases).

**Table 3:** Inter-limb power asymmetries during concentric and eccentric phases in adductor and abductor exercises.

Variable	Exercise			
	Adductor		Abductor	
	CON	ECC	CON	ECC
Mean power asymmetry (%)	15.536 ± 9.554	15.104 ± 8.020	9.296 ± 6.201	10.618 ± 5.857
Peak power asymmetry (%)	14.252 ± 9.151	17.123 ± 11.739	12.582 ± 7.826	15.928 ± 12.312

Data are presented as mean ± standard deviation (SD). CON = concentric phase; ECC = eccentric phase.

**Table 4:** Evolution of inter-limb power asymmetries in quadriceps exercises according to baseline asymmetry level. (2 groups, low and high).

Variable	Exercises							
	Quadricep Hip (Low Level)		Quadriceps Hip (High Level)		Quadriceps Knee (Low Level)		Quadriceps Knee (High Level)	
	Pre	Post	Pre	Post	Pre	Post	Pre	Post
Mean power asymmetry CON (%)	4.78 ± 3.68	5.61 ± 4.89	18.74 ± 5.86	8.23 ± 4.47 ***	6.13 ± 3.52	7.51 ± 4.74	18.28 ± 6.51	14.19 ± 5.94
	0.225; Small, *n* = 15		1.79; Large, *n* = 6		0.39; Small, *n* = 10		0.628; Moderate, *n* = 11	
Mean power asymmetry ECC (%)	5.25 ± 3.19	7.54 ± 4.77	18.25 ± 6.26	11.54 ± 11.95	5.59 ± 3.13	8.77 ± 9.79	19.11 ± 5.70	16.11 ± 7.54
	0.717; Moderate, *n* = 10		1.071; Large, *n* = 11		1.015; Large, *n* = 8		0.526; Moderate, *n* = 13	
Peak power asymmetry CON (%)	3.20 ± 2.19	5.31 ± 4.89	23.23 ± 10.31	10.74 ± 7.16 **	4.46 ± 2.86	9.17 ± 8.43	22.09 ± 8.99	18.51 ± 15.06
	0.963; Large, *n* = 13		1.211; Large, *n* = 8		1.646; Large, *n* = 11		0.398; Small, *n* = 10	
Peak power asymmetry ECC (%)	3.54 ± 2.11	5.89 ± 6.40	30.18 ± 20.75	13.36 ± 7.88	3.66 ± 3.51	2.47 ± 1.78	30.12 ± 11.66	23.21 ± 2.16
	1.156; Large, *n* = 10		0.810; Large, *n* = 11		0.339; Small, *n* = 2		0.592; Moderate, *n* = 19	

Data are presented as mean ± SD and effect size. CON = concentric phase; ECC = eccentric phase. ** *p* < 0.03, *** *p* < 0.001 (pre–post difference within the same group).

**Table 5:** Evolution of inter-limb power asymmetries in hamstring exercises according to baseline asymmetry level.

Variable	Exercises							
	Hamstring Hip (Low Level)		Hamstring Hip (High Level)		Hamstring Knee (Low Level)		Hamstring Knee (High Level)	
	Pre	Post	Pre	Post	Pre	Post	Pre	Post
Mean power asymmetry CON (%)	4.52 ± 2.94	9.30 ± 6.19	15.45 ± 5.25	11.73 ± 8.15	4.91 ± 3.01	7.32 ± 4.61	15.97 ± 6.27	13.89 ± 13.90
	1.625; Large, *n* = 13		0.708; Moderate, *n* = 8		0.800; Moderate, *n* = 13		0.331; Small, *n* = 8	
Mean power asymmetry ECC (%)	5.14 ± 3.27	10.94 ± 4.09	17.81 ± 7.20	14.42 ± 8.81	4.06 ± 2.68	6.29 ± 5.03	15.86 ± 5.57	14.70 ± 5.75
	1.773; Large, *n* = 12		0.470; Moderate, *n* = 9		0.832; Large, *n* = 14		0.208; Small, *n* = 7	
Peak power asymmetry CON (%)	3.86 ± 1.99	11.43 ± 6.24	17.78 ± 5.15	13.54 ± 12.86	3.45 ± 3.20	17.30 ± 20.27	21.81 ± 10.28	7.15 ± 3.32
	3.804; Large, *n* = 8		0.823; Large, *n* = 13		4.328; Large, *n* = 13		1.426; Large, *n* = 8	
Peak power asymmetry ECC (%)	3.43 ± 2.53	19.37 ± 13.91	24.85 ± 16.67	17.22 ± 13.78	5.14 ± 2.97	24.00 ± 31.59	35.61 ± 17.59	25.75 ± 38.78
	6.300; Large, *n* = 5		0.457; Small, *n* = 16		6.350; Large, *n* = 8		0.560; Moderate, *n* = 13	

Data are presented as mean ± SD and effect size. CON = concentric phase; ECC = eccentric phase.

**Table 6:** Evolution of inter-limb power asymmetries in adductor and abductor exercises according to baseline asymmetry level.

Variable	Exercises							
	Adductor (Low Level)		Adductor (High Level)		Abductor (Low Level)		Abductor (High Level)	
	Pre	Post	Pre	Post	Pre	Post	Pre	Post
Mean power asymmetry CON (%)	6.81 ± 3.47	12.59 ± 10.75	20.90 ± 7.95	10.52 ± 8.21	3.91 ± 2.21	7.39 ± 3.30	14.19 ± 4.13	11.20 ± 8.45
	1.665; Large, *n* = 8		1.305; Large, *n* = 13		1.574; Large, *n* = 10		0.723; Moderate, *n* = 11	
Mean power asymmetry ECC (%)	7.16 ± 1.82	13.34 ± 9.06	18.28 ± 7.27	14.21 ± 10.70	5.63 ± 2.97	10.84 ± 6.25	15.14 ± 3.64	11.67 ± 7.95
	3.395; Large, *n* = 6		0.559; Moderate, *n* = 15		1.754; Large, *n* = 10		0.953; Large, *n* = 11	
Peak power asymmetry CON (%)	4.75 ± 2.45	14.59 ± 7.11	18.05 ± 7.95	15.27 ± 11.78	6.17 ± 2.65	7.75 ± 7.08	18.40 ± 6.14	13.80 ± 9.64
	4.016; Large, *n* = 6		0.349; Small, *n* = 15		0.596; Moderate, *n* = 10		0.749; Moderate, *n* = 11	
Peak power asymmetry ECC (%)	5.43 ± 3.31	11.01 ± 5.92	22.96 ± 9.82	18.89 ± 13.18	6.43 ± 2.43	10.95 ± 6.95	23.04 ± 11.92	22.95 ± 13.01
	1.685; Large, *n* = 7		0.414; Small, *n* = 14		1.860; Large, *n* = 9		0.001; Trivial, *n* = 12	

Data are presented as mean ± SD and effect size. CON = concentric phase; ECC = eccentric phase. (pre–post difference within the same group).

**Table 7:** Results of the ANCOVA analysis.

Variable	Exercises					
	Quadricep Hip	Quadricep Knee	Hamstring Hip	Hamstring Knee	Adductor	Abductor
	F, p, η^2^, 95%CI, d	F, p, η^2^, 95%CI, d	F, p, η^2^, 95%CI, d	F, p, η^2^, 95%CI, d	F, p, η^2^, 95%CI, d	F, p, η^2^, 95%CI, d
Mean power asymmetry CON	1.517, 0.234, 0.061, 0.000–0.342, 1.082	0.049, 0.827, 0.002, 0.000–0.169, 0.152	0.544, 0.470, 0.029, 0.000–0.287, 0.573	0.739, 0.401, 0.039, 0.000–0.307, 0.620	0.907, 0.353, 0.048, 0.000–0.322, 0.630	1.354, 0.260, 0.069, 0.000–0.353, 0.961
Mean power asymmetry ECC	0.097, 0.760, 0.005, 0.000–0.206, 0.228	0.116, 0.737, 0.005, 0.000–0.207, 0.264	0.117, 0.737, 0.006, 0.000–0.213, 0.240	3.678, 0.071, 0.170, 0.000–0.464, 1.618	0.032, 0.859, 0.002, 0.000–0.162, 0.113	0.017, 0.897, 0.000, 0.000–0.136, 0.103
Peak power asymmetry CON	1.179, 0.292, 0.061, 0.000–0.343, 0.910	3.546, 0.076, 0.157, 0.000–0.452, 1.427	9.462, 0.007, 0.220, 0.000–0.509, 2.683	0.580, 0.465, 0.031, 0.001–0.292, 0.586	0.012, 0.915, 0.000, 0.000–0.120, 0.071	5.669, 0.029, 0.213, 0.000–0.503, 1.733
Peak power asymmetry ECC	6.669, 0.019, 0.255, 0.002–0.537, 1.537	0.480, 0.497, 0.026, 0.000–0.279, 0.634	0.556, 0.465, 0.029, 0.000–0.286, 0.455	0.031, 0.863, 0.002, 0.000–0.160, 0.117	0.630, 0.438, 0.024, 0.000–0.276, 0.531	0.102, 0.753, 0.003, 0.000–0.183, 0.193

CON = concentric phase; ECC = eccentric phase. CI = Confidence intervals.

## Data Availability

The original contributions presented in this study are included in the article. Further inquiries can be directed to the corresponding authors.
